# The Prognosis of Nasopharyngeal Carcinoma Involving Masticatory Muscles: A Retrospective Analysis for Revising T Subclassifications: Erratum

**DOI:** 10.1097/01.md.0000464943.60446.7f

**Published:** 2015-04-17

In the article “The Prognosis of Nasopharyngeal Carcinoma Involving Masticatory Muscles: A Retrospective Analysis for Revising T Subclassifications”,^1^ which appeared in Volume 94, Issue 4 of *Medicine*, statistical corrections were required to the Local Relapse-free Survival Rate and Regional Relapse-free Survival Rate panels of Figure 4. In addition, relevant data in Table 3 has been moved one column to the right. The article has since been corrected online.
